# Comparative Analyses of the Complete Mitogenomes of Two *Oxyria* Species (Polygonaceae) Provide Insights into Understanding the Mitogenome Evolution Within the Family

**DOI:** 10.3390/ijms252211930

**Published:** 2024-11-06

**Authors:** Lijuan Li, Zhuo Jiang, Ye Xiong, Caleb Onoja Akogwu, Olutayo Mary Tolulope, Hao Zhou, Yanxia Sun, Hengchang Wang, Huajie Zhang

**Affiliations:** 1 CAS Key Laboratory of Plant Germplasm Enhancement and Specialty Agriculture, Wuhan Botanical Garden, Chinese Academy of Sciences, Wuhan 430074, China; lilijuan18@mails.ucas.ac.cn (L.L.); jiangzhuo19991@163.com (Z.J.); xiongye23@mails.ucas.ac.cn (Y.X.); acalebonoja@mails.ucas.ac.cn (C.O.A.); olutayotolulope42@gmail.com (O.M.T.); sunyanxia@wbgcas.cn (Y.S.); 2 Center of Conservation Biology, Core Botanical Gardens, Chinese Academy of Sciences, Wuhan 430074, China; 3 University of Chinese Academy of Sciences, Beijing 100049, China; 4 Key Laboratory of Molecular Biophysics of the Ministry of Education, College of Life Science and Technology, Huazhong University of Science and Technology, Wuhan 430074, China; d201477433@alumni.hust.edu.cn

**Keywords:** *Oxyria*, Polygonaceae, mitogenomes, phylogenetic analysis, repeat-mediated recombination

## Abstract

*Oxyria* (Polygonaceae) is a small genus only comprising two species, *Oxyria digyna* and *O. sinensis*. Both species have well-documented usage in Chinese herbal medicine. We sequenced and assembled the complete mitogenomes of these two species and conducted a comparative analysis of the mitogenomes within Polygonaceae. Both *O. digyna* and *O. sinensis* displayed distinctive multi-branched conformations, consisting of one linear and one circular molecule. These two species shared similar gene compositions and exhibited distinct codon preferences, with mononucleotides as the most abundant type of simple sequence repeats. In the mitogenome of *O. sinensis*, a pair of long forward repeat sequences can mediate the division of molecule 1 into two sub-genomic circular molecules. Homologous sequence analysis revealed the occurrence of gene transfer between the chloroplast and mitochondrial genomes within *Oxyria* species. Additionally, a substantial number of homologous collinear blocks with varied arrangements were observed across different Polygonaceae species. Phylogenetic analysis suggested that mitogenome genes can serve as reliable markers for constructing phylogenetic relationships within Polygonaceae. Comparative analysis of eight species revealed Polygonaceae mitogenomes exhibited variability in gene presence, and most protein-coding genes (PCGs) have undergone negative selection. Overall, our study provided a comprehensive overview of the structural, functional, and evolutionary characteristics of the Polygonaceae mitogenomes.

## 1. Introduction

*Oxyria* is a small genus in the Polygonaceae, consisting of two species, *Oxyria digyna* (L.) Hill and *O. sinensis* Hemsley [1]. *Oxyria digyna* thrives in various moderately humid tundra habitats and is widely distributed across the Northern Hemisphere. It is highly adapted to high-altitude environments, reaching elevations up to ca. 4900 m [1,2]. In contrast, *O. sinensis* is native to China, predominantly grows in the middle to high-altitude regions of southwestern mountains of China like Yunnan, Sichuan, and Xizang (Tibet), ranging from 1600 to 3800 m [3,4]. *Oxyria sinensis* is dioecious and typically grows in resource-poor habitats, such as slopes and valleys. They are often found in copper-zinc mining areas and serve as indicator plants for copper deposits, earning them the nickname “copper weed” [1,5]. All wild records showed *Oxyria* species have demonstrated remarkable adaptation to harsh environmental conditions, such as cold temperatures, high altitudes, and nutrient-poor soils. Both *O. digyna* and *O. sinensis* have been used as medicinal herbs for their anti-inflammatory, cathartic, antimicrobial, and antidiarrheal properties [1,6]. The strong ecological adaptative ability and diverse natural compounds made *O. digyna* and *O. sinensis* excellent candidates for investigating the genetic information, which is associated with genome evolution and natural compound synthesis within the Polygonaceae. While the chloroplast genomes of *Oxyria* have been published [7], investigation on their mitogenomes remains blank.

The Polygonaceae is globally distributed and particularly abundant in the temperate regions of the Northern Hemisphere [8]. The family comprises approx. 50 genera and more than 1000 species [7]. Approximately 13 genera and more than 200 species are distributed in China. Many species within the family have important economic values. For example, buckwheat (*Fagopyrum esculentum*) is a crucial grain crop [9], and *Polygonum orientale* and *P. capitata* are introduced as ornamental plants. Also, some species, such as *Pleuropterus multiflorus*, *Reynoutria japonica*, *Rheum palmatum*, and *Fallopia aubertii*, are widely used in traditional Chinese medicine [10,11,12,13]. The chloroplast genomes of multiple species within the Polygonoideae have been assembled and reported for phylogenetic, evolutionary, and horticultural studies [7,14]. The entire nuclear genomes of some species with high commercial and ecological value, such as *F. esculentum*, *Reynoutria japonica*, *Rheum officinale*, and *Rheum nobile* have been studied [15,16,17,18].

The mitogenomes, typically consisting of circular or linear DNA, are crucial for metabolic processes like respiration and photosynthesis, which significantly impact the growth and development of organisms [19,20,21,22,23]. Mitochondria exhibited considerable genetic diversity, largely attributed to their high frequency of recombination. Investigating the diverse and complex nature of plant mitogenomes meets significant challenges but offers opportunities for improving our understanding in the field of evolutionary biology [21]. Many phylogenetic and molecular identification studies on Polygonaceae have been conducted using transcriptomes or chloroplast genomes [7,14]. However, to date, only six mitogenomes in the Polygonaceae have been reported. They are, *Rheum rhabarbarum*, *Rheum palmatum*, *Pleuropterus multiflorus*, *Reynoutria japonica*, *Fallopia aubertii*, and *Fagopyrum esculentum* [9,10,11,12,13]. Previous studies have shown that plant mitogenomes exhibit rich diversity in size and structure, even among closely related species [24,25]. In the six published species of Polygonaceae, mitogenome sizes ranged from 302,229 bp in *Reynoutria japonica* to 404,063 bp in *Fagopyrum esculentum* [9,11]. These mitogenomes are exclusively composed of circular structures, with variations in the number of circular chromosomes: one in *Fallopia aubertii*, two in *Rheum palmatum*, three in *Rheum rhabarbarum*, and ten in *Fagopyrum esculentum* [9,10,13]. Despite these findings, comparative analyses among the mitogenomes of Polygonaceae remain limited, requiring further investigation into mitochondrial diversity within this family.

In this study, we focused on assembling and comparatively analyzing the mitogenomes of *O. digyna* and *O. sinensis* to examine features like codon usage, sequence repeats, and gene migration events from the chloroplast to the mitogenomes. Furthermore, we compared the reported mitogenomes of some members of the Polygonaceae to identify their general characters. This study also explored the effectiveness of utilizing mitogenome data for phylogenetic inference within Polygonaceae. Our aim is to provide comprehensive insights into mitogenome evolution within Polygonaceae.

## 2. Results

### 2.1. Basic Features of the Oxyria Mitogenomes

**Figure 1:**
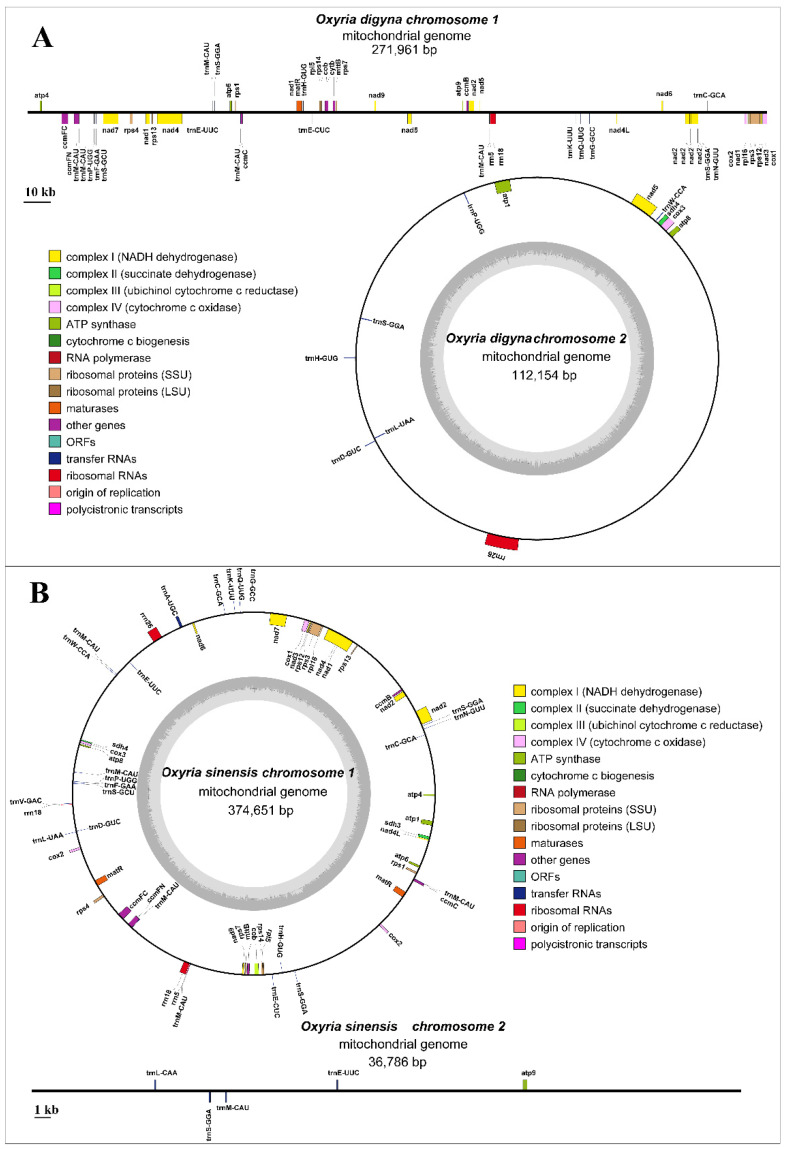
*O. digyna* (**A**) and *O. sinensis* (**B**) mitogenome gene map. Genes shown on the outside and inside of the circle are transcribed clockwise and counterclockwise, respectively. The dark grey region within the inner circle represents the GC content.

The mitogenome of *O. digyna* had a total length of 384,115 bp. It consisted of two main structures: a linear molecule (molecule 1: 271,961 bp) and a circular molecule (molecule 2: 112,154 bp), with GC contents of 45.0% and 44.4%, respectively (Figure 1A). In comparison, the mitogenome of *O. sinensis* measured 411,437 bp in total length. Also, it exhibited a multi-branched structure, consisting of a circular molecule (molecule 1, 374,651 bp) and a linear molecule (36,786 bp), with GC contents of 44.5% and 42.0%, respectively (Figure 1B). The functional categorization and physical locations of the annotated genes were displayed (Figure 1).

We annotated 34 and 36 PCGs in the mitogenomes of *O. digyna* and *O. sinensis*, respectively (Figure 1; Table 1). Both species shared the same types of PCGs (34 types) and three rRNA genes. In *O. digyna*, all PCGs were present as single copies, whereas in *O. sinensis*, the *cox*2 and *mat*R genes were in duplicates (Figure 1; Table 1). Additionally, 25 tRNA genes were annotated in *O. digyna* (with *trn*L-CAA and *trn*V-GAC absent), while 28 were annotated in *O. sinensis*. Both species had multiple copies of *trn*M-CAU and *trn*S-GGA. *O. digyna* had five copies of *trn*M-CAU and three copies of *trn*S-GGA, while *O. sinensis* had six copies of *trn*M-CAU and three copies of *trn*S-GGA (Figure 1; Table 1). Additionally, *O. digyna* had three copies of *trn*P-UGG, whereas *O. sinensis* had three copies of *trn*E-UUC. The overall gene composition of the two mitogenomes appears to be highly conserved.

### 2.2. Mitochondrial Repetitive Sequences

**Figure 2:**
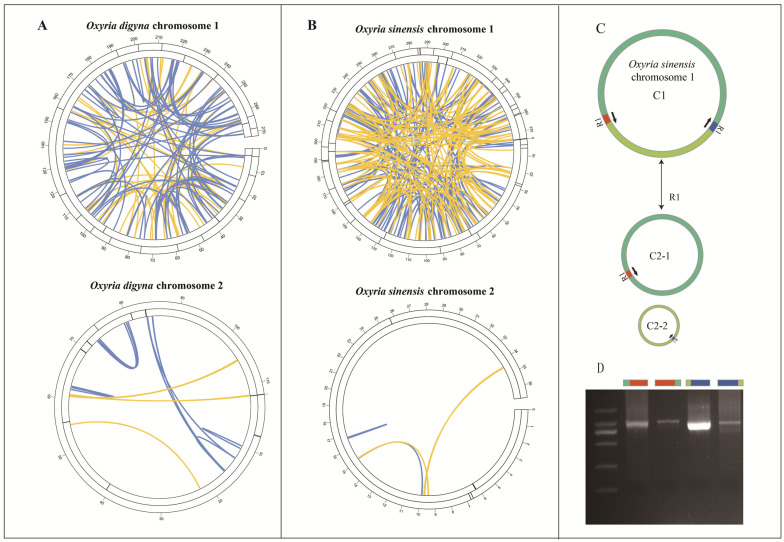
*O. digyna* (**A**) and *O. sinensis* (**B**) mitogenome repeated sequence diagram. The colored lines in inner circle shows the dispersed repeats with a length greater than or equal to 50 bp in which blue represents forward repeats and yellow represents palindromic repeats. The two outer circles show tandem repeats and simple sequence repeats, both represented as short bars. (**C**) The long repeat sequence in the mitogenome of *O. sinensis* molecule 1 mediates the potential conformations generated from the recombination. (**D**) The gel electrophoresis results of PCR products were amplified using primers. The amplified sequences correspond to the four sites of the recombination conformation. For repeat sequences that were too long to be fully amplified by PCR, we selected portions of the repeat sequences along with 200 bp of their flanking regions for the PCR experiments.

In the *O. digyna* mitogenome, we identified 16 simple sequence repeats (SSRs) in molecule 1 (linear) and 3 SSRs in molecule 2 (circular) (Figure 2A; Tables S1 and S2). In molecule 1, the majority of SSRs were monomeric. Among these monomeric SSRs, thymine (T) repeats were the most prevalent, followed by adenine (A) repeats (Table S1). Additionally, molecule 1 contained one trinucleotide SSR of the GAA type. In molecule 2, all SSRs were monomeric, comprising two thymine (T) repeats and one adenine (A) repeat (Table S2). For the *O. sinensis* mitogenome, 28 SSRs were found in molecule 1 (circular) and 3 SSRs in molecule 2 (linear) (Figure 2B; Tables S3 and S4). In molecule 1, most SSRs were also monomeric. These monomeric SSRs consisted solely of adenine (A) repeats (15 repeats) and thymine (T) repeats (11 repeats). Additionally, two SSRs in molecule 1 were dimeric, with AT and TC repeats, and one of each type (Table S3). In molecule 2, all SSRs were monomeric, consisting of two cytosine (C) repeats and one adenine (A) repeat (Table S4).

In *O. digyna*, six tandem repeats were found in both molecule 1 and molecule 2 (Figure 2; Tables S5 and S6). In *O. sinensis*, 12 tandem repeats were identified in chromosome 1, while chromosome 2 contained only one tandem repeat with a consensus size of 17 bp (Figure 2; Tables S7 and S8). We identified 181 pairs of dispersed repeats in *O. digyna* (156 in molecule 1 and 25 in molecule 2) and 267 pairs in *O. sinensis* (262 in molecule 1 and 5 in molecule 2) (Tables S9–S12). The repeats were predominantly forward and palindromic (Figure 2). In chromosome 1 of *O. digyna*, we identified 63 pairs of palindromic repeats with the longest 156 bp in length and 93 pairs of forward repeats. In chromosome 2 of *O. digyna*, six pairs of palindromic repeats (up to 136 bp) and 19 pairs of forward repeats were found. For *O. sinensis*, chromosome 1 contained 113 pairs of palindromic repeats and 149 pairs of forward repeats, with the longest extending up to 17,960 bp. In chromosome 2 of *O. sinensis*, three pairs of palindromic repeats and 2 pairs of forward repeats were identified. Our study further identified that the longest forward repeat sequences in *O. sinensis* could mediate the formation of two circular molecules in molecule 1 of the mitogenome (Figure 2C). PCR experiments validated these findings, as the length of the PCR products matched the expected results (Figure 2D).

### 2.3. Codon Usage Preference

**Figure 3:**
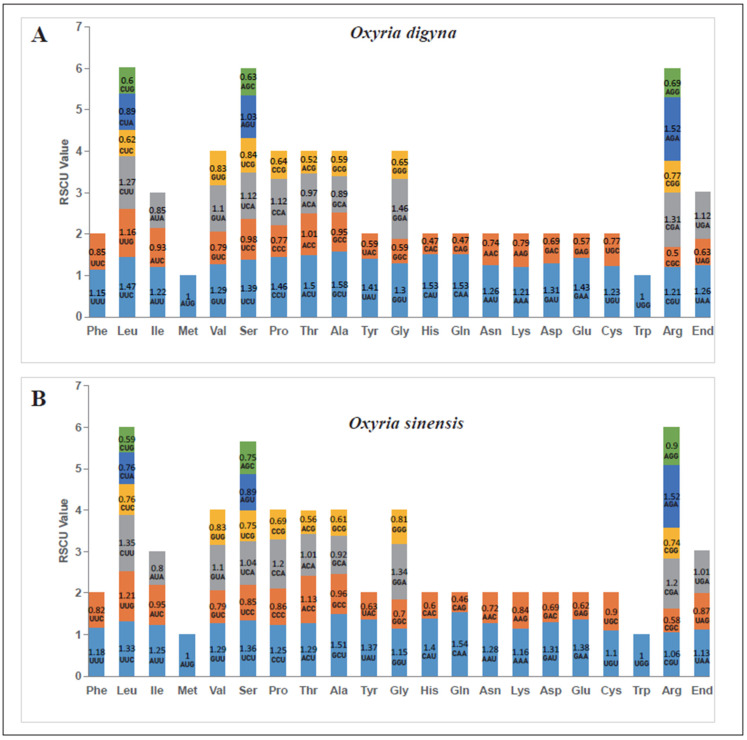
*O. digyna* (**A**) and *O. sinensis* (**B**) mitogenome relative synonymous codon usage. The codon families are shown on the *X*-axis. The RSCU values indicate how frequently a specific codon is observed compared to its expected frequency under uniform synonymous codon usage. RSCU values greater than 1 suggest a preference for specific amino acids in codon usage.

The codon usage analysis was performed on the 34 PCGs in the mitogenomes of *O. digyna* and *O. sinensis*, respectively (Figure 3). The RSCU values for the start codon AUG (Met) and UGG (Trp) for both species were consistently 1, due to the absence of alternative synonymous codons. A general trend of codon preference was observed in the mitochondrial PCGs of both *O. digyna* (A) and *O. sinensis* (B). In both species, there were 30 codons with RSCU values greater than 1, of which 27 ended in A or U, accounting for 90%. Conversely, there were 32 codons with RSCU values less than 1, with 28 of them ending in G or C bases, making up 87.5%. Among all amino acid codons, those encoding Leucine (Leu), Serine (Ser), and Arginine (Arg) were the most abundant in both *O. digyna* and *O. sinensis*. In *O. digyna*, the RSCU values exceeding 1.5 included GCU (Ala), CAU (His), CAA (Gln), AGA (Arg), and ACY (Thr), with GCU (Ala) having the highest RSCU value of 1.58 in mitochondrial PCGs. Conversely, in *O. sinensis*, only CAA (Gln), AGA (Arg), and GCU (Ala) had RSCU values above 1.5, with CAA (Gln) showing the highest RSCU value of 1.54 in mitochondrial PCGs (Figure 3).

### 2.4. Characterization of Chloroplast Genome Transfer into the Mitogenome

**Figure 4:**
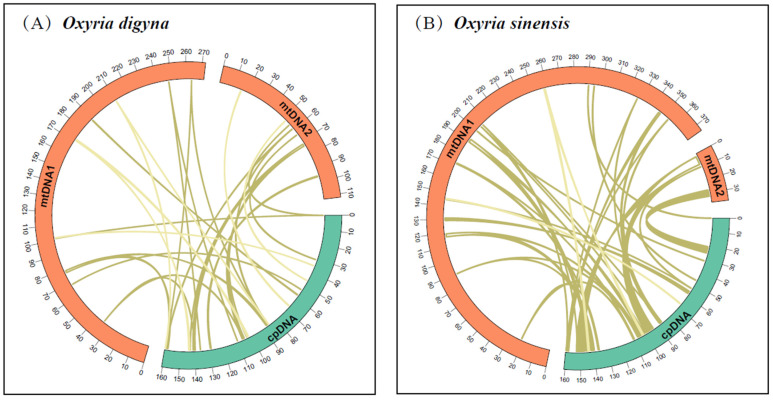
Schematic diagram of gene transfer between chloroplast and mitogenomes in *O. digyna* (**A**) and *O. sinensis* (**B**). The orange and green arcs represent the mitogenome and chloroplast genomes, respectively, with the yellow lines between the arcs corresponding to homologous genomic fragments.

We identified 27 and 33 homologous fragments between the mitochondrial and chloroplast genomes in *O. digyna* and *O. sinensis*, respectively (Figure 4). In *O. digyna*, the total length of homologous fragments was 10,585 bp, constituting 2.76% of the mitogenome length. Notably, 5 of the 27 homologous fragments exceeded 1000 bp, with fragments 1 and 2 being the longest at 1439 bp (Table S13). In *O. sinensis*, the total length of the homologous fragments was 34,819 bp, comprising 8.46% of the mitogenome length. Ten of the 33 homologous fragments exceeded 1000 bp, with fragments 1 and 2 the longest at 6104 bp (Table S14). Annotation of these homologous sequences found 13 complete genes within the 27 homologous fragments of the *O. digyna* mitogenome, including ten complete tRNA genes (one *trn*D-GUC, two *trn*H-GUG, three *trn*M-CAU, two *trn*N-GUU, one *trn*S-GGA, and one *trn*W-CCA) and three PCGs (one *pet*G and two *rpl*23). In the *O. sinensis* mitogenome, 17 complete genes were annotated in 33 homologous fragments, including eight PCGs (two *ndh*B, two *rps*7, one *rpo*C1, one *rbc*L, and two *rpl*23) and nine tRNA genes (two *trn*A-UGC, two *trn*I-CAU, one *trn*L-CAA, one *trn*M-CAU, two *trn*V-GAC, and one *trn*W-CCA).

### 2.5. Phylogeny and Synteny

**Figure 5:**
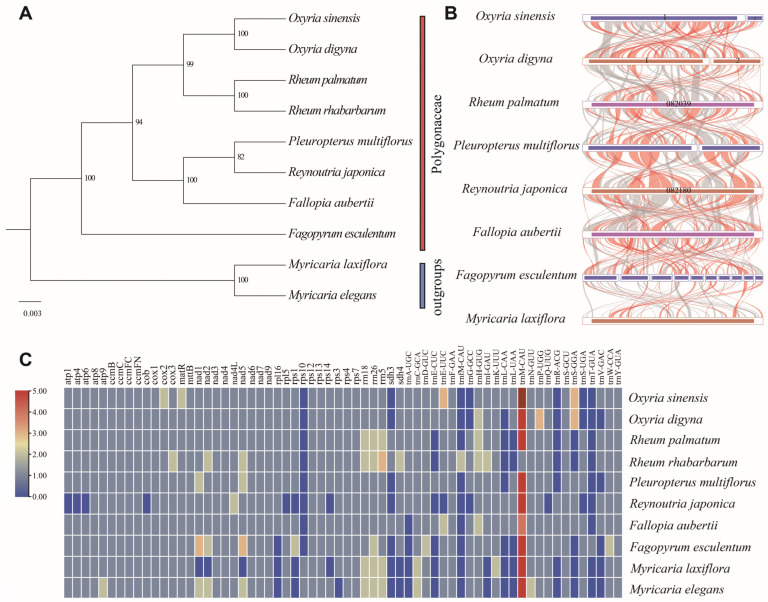
The phylogenetic relationships (**A**) and mitogenomes synteny (**B**) and mitochondrial genes distribution (**C**) of *O. digyna* and *O. sinensis* with the 8 closely related species. The Maximum Likelihood tree was constructed based on the sequences of 20 conserved PCGs. Regarding mitogenome synteny, bars indicate the mitogenomes, and the ribbons display the homologous sequences between the adjacent species. The red areas indicate where the reversal occurred; the grey areas indicate regions of good homology. Common blocks less than 500 bp in length are not retained, and regions without a common block indicate that they are peculiar to the species. Regarding mitochondrial gene distribution, the colors of the boxes indicate the number of copies that exist in the mitogenomes.

The phylogenetic tree was constructed using 20 conserved mitochondrial PCGs (*atp*8, *atp*9, *ccm*B, *ccm*C, *ccm*FC, *ccm*FN, *cox*1, *cox*2, *cox*3, *mat*R, *mtt*B, *nad*3, *nad*4L, *nad*6, *nad*9, *rps*12, *rps*13, *rps*3, *rps*4, and *rps*7) from the ten species. The phylogenetic tree showed strong support for all clades and nodes, particularly for the sister relationship between *O. digyna* and *O. sinensis*. The *Oxyria* clade was sister to *Rheum* (Figure 5A), congruent with the previous study [7,14]. Pairwise synteny analysis revealed a large number of homologous collinear blocks that were not arranged in the same order across the species (Table S15; Figure 5B). The highest abundance of short homologous sequences was observed between the mitogenomes of *Fallopia aubertii* and *Fagopyrum esculentum*, despite these species belonging to different genera. The mitogenomes of *O. digyna* and *O. sinensis* contained a significantly higher number of homologous sequences compared to those of other species. Conversely, the fewest homologous sequences were identified between *Pleuropterus multiflora* and *Reynoutria japonica*. However, they contained much longer homologous fragments.

### 2.6. Variation in Gene Composition in Mitogenomes of Polygonaceae

We compared the distribution of mitochondrial genes among species in Polygonaceae (Figure 5C), which revealed a high diversity in their gene composition. The *trn*M-CAU gene was present in multiple copies across all species within the Polygonaceae. In contrast, the *rps*10, *trn*fM-CAU (present only in *Rheum rhabarbarum*), and *trn*T-GUA genes (found exclusively in *Fagopyrum esculentum*) were mostly absent in other species. Among the ten species examined, *Rheum rhabarbarum* possessed the highest number of mitochondrial genes (68), while *Reynoutria japonica* had the fewest (41). Both *O. digyna* and *O. sinensis* exhibited the loss of the same PCGs (*rps*10 and *sdh*3). Additionally, *Reynoutria japonica* showed the highest gene loss, and the missing genes are *atp*1, *atp*4, *atp*6, *cob*, *rpl5*, *rps*1, *rps*10, *rps*14, and *sdh*3.

### 2.7. Variation in Substitution Rates of Mitochondrial PCGs

**Figure 6:**
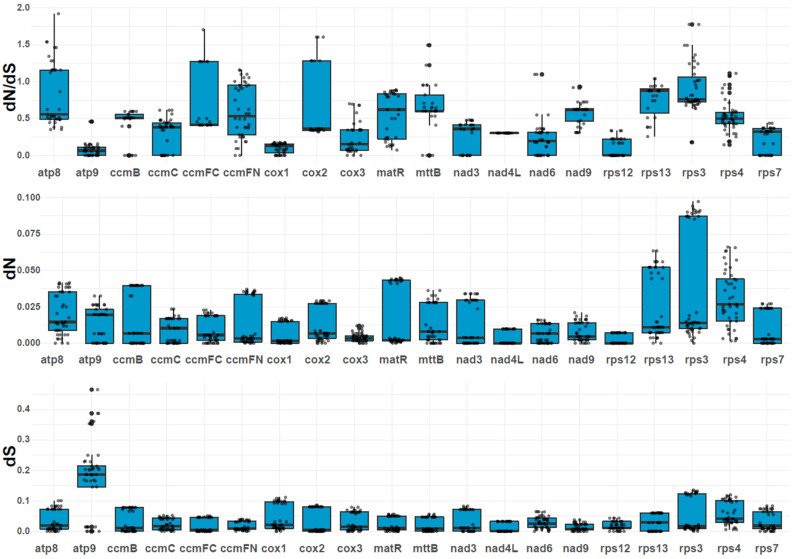
Boxplots of pairwise dN, dS values and their ratio among 20 mitochondrial genes in *O. digyna* and *O. sinensis* and 8 closely related species mitogenomes.

We estimated the pairwise nucleotide substitution rates, including the nonsynonymous substitution rate (dN), synonymous substitution rate (dS), and the dN/dS ratio (Figure 6; Table S16). The dN/dS values for most genes were less than 1.0, accounting for 50% of the 20 PCGs analyzed (Figure 6). The 10 genes are *atp*9, *ccm*B, *ccm*C, *cox*1, *cox*3, *mat*R, *nad*3, *nad*4L, *rps*12, and *rps*7. Among these genes, *atp*9 and *cox*1 had the lowest dN/dS values (dN/dS < 0.25), suggesting that they have undergone strong purifying selection. The dN/dS values were consistently conserved across the studied species. However, some genes, such as *atp*8, *ccm*FC, *ccm*FN, *cox*2, *mtt*B, *nad*6, *rps*13, *rps*3, and *rps*4, showed dN/dS values greater than 1, indicating that these genes may have been subjected to positive selection during evolution. Notably, the *atp*8 gene exhibited an exceptionally high dN/dS value (e.g., *O. sinensis* vs. *Fallopia aubertii*: 1.92), suggesting strong positive selection pressure.

## 3. Discussion

### 3.1. General Features of the Oxyria Mitogenomes

In our study, mitogenomes of the *Oxyria* were sequenced and assembled for the first time. We discovered that both *O. digyna* and *O. sinensis* exhibit notable structural polymorphisms characterized by the presence of both linear and circular molecules. This is distinct from the mitochondrial structures reported in other reported Polygonaceae species, which typically have only circular molecules (one circular chromosome in *Fallopia aubertii*, two in *Rheum palmatum*, three in *Rheum rhabarbarum*, and ten in *Fagopyrum esculentum*) [9,10,13]. This structural variation may be considered as a phylogenetic signature in the independent evolution of *Oxyria* in Polygonaceae. A similar genome conformation has also been reported in other plants. For example, the mitogenome of *Mentha spicata* consists of a linear chromosome and two circular chromosomes [26]. Such structural variability is not rare across various plant species and is likely influenced by the presence of repetitive sequences. Generally, a higher level of structural variability corresponds to a greater number of repetitive sequences. The gene composition in *Oxyria* species is generally similar, although *O. digyna* has lost the *trn*L-CAA and *trn*V-GAC. This suggests that while the PCGs are highly conserved, tRNA genes demonstrated greater variability within the mitogenomes of the genus *Oxyria*. These findings may contribute to our understanding of the diversity of mitogenome structures within Polygonaceae.

Small repeat sequences are crucial determinants of the size of plant mitogenomes [23,27,28]. The size of the *O. digyna* or *O. sinensis* mitogenome is comparable to that of other Polygonaceae species with reported mitogenomes but larger than that of *Fagopyrum esculentum* [9]. Notably, the mitogenome of *O. sinensis* is the longest among them, featuring a pair of long repetitive sequences spanning 17,960 bp, which resulted in the duplication of the *cox*2 and *mat*R genes. A similar phenomenon is observed in *Taraxacum mongolicum*, which contains a pair of 21,809 bp repeat sequences [29]. As active sites of recombination, these repeat sequences may have significantly influenced the structure of plant mitogenomes, leading to diverse forms such as circular, linear, or branched molecules. In this study, we identified 19 and 31 SSRs in the mitogenomes of *O. digyna* and *O. sinensis*, respectively. Notably, mononucleotide repeats were the most prevalent type of SSRs in both *O. digyna* and *O. sinensis*. Most monomeric SSRs consisted of A and T bases rather than G and C, consistent with the observations in *Rheum palmatum* [10] and *Cymbidium ensifolium* [30]. This bias may be attributed to the lower energy requirement to break A–T bonds compared to G–C bonds. Long repeat sequences also play a critical role in mediating homologous recombination in plant mitogenomes [19,23]. The repeat sequences analysis indicated a considerable diversity of tandem repeats and dispersed repeats between *O. digyna* and *O. sinensis*. Repetitive sequences in mitogenomes can serve as recombination sites, contributing to the formation of multiple molecular conformations. In our study, a long pair of repeat sequences can mediate the formation of two independent circular molecules. Our results provided additional evidence for the presence of multiple conformations within the mitogenome of plants.

Codon bias analysis can provide inferences into horizontal gene transfers and evolutionary relationships among organisms, as closely related species often display similar codon usage patterns [31,32]. The codon usage analysis of PCGs in the mitogenomes of *O. digyna* and *O. sinensis* revealed a preference for A/U-ending codons, which is consistent with the patterns observed in *Rheum palmatum* [10] and *Reynoutria japonica* [11]. This preference may reflect an evolutionary adaptation for maintaining cellular equilibrium. Both *Oxyria* species showed a higher usage of codons encoding leucine (Leu), serine (Ser), and arginine (Arg), with specific codons like GCU (Ala), CAU (His), AGA (Arg), and CAA (Gln) notably prevalent. This bias towards certain codons and amino acids may influence mitochondrial gene expression and the efficiency of protein synthesis.

### 3.2. Intergenomic Sequence Transfers

Chloroplasts and mitochondria are semi-autonomous organelles of endosymbiotic origin [33,34]. Horizontal gene transfer frequently occurs between plastids and mitochondria, resulting in DNA exchange from plastid to mitochondria (MTPT) [23,35,36,37,38]. In this study, we identified MTPT fragments 10,585 bp in *O. digyna* and 34,819 bp in *O. sinensis*, which constituted 2.76% and 8.46% of their respective mitogenome lengths. Comparatively, the MTPT homologous sequence lengths in other Polygonaceae species, such as *Fallopia aubertii* and *Reynoutria japonica*, were found to be 47,757 bp and 26,123 bp, accounting for 13.6% and 8.64% of their mitogenomes, respectively. Our results revealed that MTPTs are particularly common, and their abundance varies significantly among different species in Polygonaceae. In the mitogenomes of *O. digyna* and *O. sinensis*, ten and nine complete homologous tRNA gene fragments were found. A similar phenomenon was also presented in *Fallopia aubertii* and *Reynoutria japonica* [11,13]. The three complete MTPT genes (*trn*A-UGC, *trn*L-CAA, and *trn*V-GAC) detected in *O. sinensis* were also found in *Fallopia aubertii* and *Reynoutria japonica* [11,13]. The six genes, including *trn*H-GUG, *trn*M-CAU, *trn*N-GUU, *trn*W-CCA, *trn*P-UGG, and *trn*S-GGA are commonly recognized as MTPT genes in angiosperms [39], and our study reaffirms this observation. MTPTs played a crucial role in the precise assembly of chloroplast and mitochondrial genomes. The sequence transfer analysis enhanced our understanding of the evolution of organelle genomes.

### 3.3. Phylogenetic Inference and Synteny Analysis

The mitogenome, with its advantages such as maternal inheritance, rapid evolution, and low recombination rates, has become a valuable tool for taxonomy, phylogeny, evolution, and population genetics studies [19,40]. The phylogenetic relationships revealed by our analysis were consistent with previous studies [7,8,9,10,11,12,13]. This consistency may have underscored the reliability of mitochondrial genes in constructing phylogenetic relationships within Polygonaceae. The mitogenomes of O. digyna and O. sinensis can serve as informative resources for further phylogenetic studies in Polygonaceae. With rapid advancements in sequencing technology, an increasing number of complete plant mitogenomes have been assembled and reported, thus facilitating comparative analyses of mitogenome characteristics across various species [21,40]. Pairwise synteny analysis suggested that mitogenomes within Polygonaceae have undergone extensive rearrangements. They also exhibited a high degree of structural non-conservation, which may play a crucial role in the evolution and diversification of plant mitogenomes.

### 3.4. Comparative Analysis of Gene Composition and Selective Pressure Analysis

The mitogenome composition in Polygonaceae exhibited a mix of conservation and variability among PCGs, rRNA, and tRNA, reflecting evolutionary adaptations and functional requirements. Common PCGs are highly conserved and are essential for mitochondrial functions. However, *O. digyna* and *O. sinensis* have lost some PCGs, specifically *rps*10 and *sdh*3, which are involved in the biosynthesis of ribosomal proteins and succinate dehydrogenase, respectively. Similarly, *Reynoutria japonica* has been reported to have the most gene losses among the species studied. Although it retains all NADH dehydrogenase-related genes [11]. Previous studies have proposed that some mitochondrial genes appear lost or transferred to the nuclear genome, with chloroplast genes potentially compensating for these losses [35,36]. The extensive intergenomic sequence transfers observed may have contributed to the evolutionary dynamics and functional adaptations of mitogenomes within Polygonaceae [11,41]. The universal presence of rRNA genes (*rrn*18, *rrn*26, and *rrn*5) emphasized their critical role in mitochondrial ribosome function. While tRNA genes are broadly represented, there are notable variations. For example, high copies of *trn*M-CUA were detected across all species in the Polygonaceae. Conversely, *trn*fM-CAU (present only in *Rheum rhabarbarum*) and *trn*T-GUA (present only in *Fagopyrum esculentum*) were nearly absent in other species within the family, indicating possible gene loss or reduced functional necessity.

Substitutions at synonymous and nonsynonymous sites help differentiate between neutral and selective forces acting on genes [42,43,44]. The analysis of nucleotide substitution rates among mitochondrial PCGs showed most genes had a dN/dS ratio less than 1.0, indicating purifying selection. This result was consistent with previous studies [11,13,45,46], revealing that most mitochondrial genes are conserved. Genes such as *atp*9 and *cox*1 exhibited particularly low dN/dS values, suggesting strong conservation across the mitogenomes of Polygonaceae. This conservation reflects the essential roles of these genes in mitochondrial function and the selective pressures to maintain their integrity. The dN/dS values of protein-coding genes such as *atp*8, *ccm*FC, *ccm*FN, and *cox*2 were found to be larger than 1. These three genes may have experienced positive selection due to environmental stress, leading to the development of new functions to adapt to the changing environment.

## 4. Materials and Methods

### 4.1. Sample Collection and DNA Sequencing

The sample of *O. sinensis* was collected from a plant growing in the Wuhan Botanical Garden (Wuhan, China), which was originally introduced from Shade Town, Kangding City, Sichuan Province, southwestern China (longitude: 101.3334, latitude: 29.5552). The sample of *O. digyna* was collected from the Changbai Mountain in Antu County, Yanbian Korean Autonomous Prefecture, Jilin Province, northeastern China (longitude: 128.0575, latitude: 42.0453). Fresh leaves of the two species were collected, immediately frozen in liquid nitrogen, and stored at −80 °C. High-quality genomic DNA was extracted using the CTAB method [47]. We utilized both Illumina short-read sequencing and Oxford Nanopore long-read sequencing. The short-paired reads were sequenced by Illumina HiSeq X ten (Illumina, Inc.; San Diego, CA, USA). For Oxford Nanopore sequencing, a 10 kb insert size library was prepared and sequenced on the Nanopore PromethION platform (Oxford Nanopore Technologies, Oxford, UK). We employed NanoPack v2 to assess and ensure the quality of the raw reads [48].

### 4.2. Mitogenome Assembly and Annotation

We assembled the mitogenomes of the two *Oxyria* species using a hybrid approach that combined Illumina short reads with long reads from Oxford Nanopore sequencing. Initially, a de-novo assembly was performed using SMARTdenovo software v3.0 with Nanopore long reads [49]. Predicted mitochondrial contigs were then filtered against the mitogenome reference of *Rheum palmatum* (accession number: OR148905). The selected contigs were polished for three iterations using Miniasm v0.3 and Racon v1.5.0 [50,51]. Mitochondrial sequences from Illumina short reads were filtered by mapping the clean short reads to the predicted mitochondrial contigs using Bowtie2 and SAMtools v1.6 [52,53]. The final assembly of the mitogenome was conducted by combining the filtered Illumina short reads and Nanopore long reads with Unicycler v0.5.0 [54]. The assemblies were visualized using Bandage v0.9.0 [55], with GFA format files generated by Unicycler v0.5.0 [54]. Additionally, the chloroplast genomes of *Oxyria* were assembled using GetOrganelle v1.7.7.0 [56] with the parameters “-R 10 -t 1 -k 21,45,65,85,105”.

We annotated the mitogenomes of two *Oxyria* species using the GeSeq web server (https://chlorobox.mpimp-golm.mpg.de/geseq.html, accessed on 7 March 2024) [57], with the mitogenome of *R. palmatum* mentioned above as the reference. Transfer RNAs (tRNAs) and ribosomal RNAs (rRNAs) were identified using tRNAscan-SE v2.0.12 [58]. The circular maps of the mitogenomes were generated with OGDraw v1.0 [59]. The chloroplast genomes were annotated using Geneious v9.0.2 [60] and GeSeq (https://chlorobox.mpimp-golm.mpg.de/geseq.html, accessed on 7 March 2024). All annotations of both mitochondrial and chloroplast genomes were thoroughly reviewed and manually corrected using Apollo v2.5.0 [61].

### 4.3. Analysis of Repetitive Sequence and Repeat-Mediated Homologous Recombination

We identified three types of repeats: simple sequence repeats (SSRs), tandem repeats, and dispersed repeats. SSRs were identified using MISA-web (https://webblast.ipk-gatersleben.de/misa/, accessed on 7 March 2024) with the following parameters: 1–10, 2–5, 3–4, 4–3, 5–3, and 6–3 [62]. Tandem repeats were detected using Tandem Repeats Finder v4.09 (http://tandem.bu.edu/trf/trf.submit.options.html, accessed on 7 March 2024) with the parameters: match +2, mismatch –7, and indel –7 [63]. Dispersed repeats, including forward (F), reverse (R), palindromic (P), and complement (C) repeats, were identified using the online version of REPuter (https://bibiserv.cebitec.uni-bielefeld.de/reputer/, accessed on 7 March 2024). The search criteria included a minimum repeat size of 30 bp and a repeat identity greater than 90%, with the following parameters: Hamming distance of 3, a maximum of 50 computed repeats, and a minimum repeat size of 8 [64]. The results of the repeat analysis of the mitogenomes were visualized using the Circos package, which was implemented in TBtools v2 [65].

We identified potential structures of circular molecules in the mitogenome using long-read data. Specifically, we extracted the repetitive sequences and their flanking regions (1000 bp) from the mitogenome, representing sequences that support the master structure. We then swapped the flanking regions to represent sequences that might support possible recombination. These major and recombinant sequences were mapped to the long reads using BLASTN v2.4 [66] to determine the presence of recombination sequences. To verify the reliability, we performed a PCR experiment using primers designed based on the repeat sequences and their upstream and downstream regions. The PCR reaction mixture consisted of 1 μL DNA, 1 μL of 10 μM forward and reverse primers each, 13 μL of 2× Taq PCR Master Mix, and 10 μL of ddH_2_O. The PCR conditions were as follows: initial denaturation at 94 °C for 3 min; 30 cycles of 94 °C for 30 s, 55 °C for 30 s, and 72 °C for 1 min, followed by a final extension at 72 °C for 10 min.

### 4.4. Analysis of Codon Preference and Chloroplast to Mitochondrion DNA Transformation

PhyloSuite v1.2.2 was used to extract protein-coding genes (PCGs) from the mitogenomes [67]. Codon preference analysis of the mitochondrial PCGs was then performed using MEGA XI [68], and relative synonymous codon usage (RSCU) values were calculated. To identify homologous sequences between the chloroplast and mitogenomes, we assembled the chloroplast genomes of *O. digyna* and *O. sinensis* using short reads with GetOrganelle v1.7.7.0 [56]. Homologous sequences were identified using BLASTN software v2.4 [66] with an e-value cut-off of 1 × 10^−5^. The distribution of mitochondrial plastid sequences (MTPTs) across all genomes was visualized using the Circos package [69] implemented in TBtools v2 [65].

### 4.5. Phylogenetic and Syntenic Analysis

To determine the phylogenetic position of *Oxyria* species within Polygonaceae, we integrated the mitogenomes of six published Polygonaceae species from five genera (*Rheum*, *Pleuropterus*, *Reynoutria*, *Fallopia*, and *Fagopyrumn*). *Myricaria laxiflora* and *Myricaria elegans,* belonging to the Tamaricaceae family within the order Caryophyllales, were used as outgroups. The mitogenome sequences were downloaded from NCBI (Table S17). The shared genes from the mitochondrial genomes were identified, extracted, and concatenated using PhyloSuite v1.2.2 [67]. Multiple sequence alignment was performed with MAFFT v7.4 [70]. A maximum likelihood (ML) phylogenetic tree was constructed based on shared PCGs using MEGA XI software [68], applying the most commonly used GTR+G+I nucleotide substitution model with 1000 bootstrap replicates. Additionally, we conducted a comparative analysis of the gene composition of the mitogenomes. The results were visualized using heatmap plots generated with the *ggplot*2 package in R v4.2 [71]. Pairwise synteny analysis of mitogenomes among *Oxyria* and six other species was performed using BLASTN v2.4 [66]. This analysis involves identifying conserved collinear segments, specifically focusing on homologous sequences longer than 500 bp. The visualization of these homologous segments was accomplished using the Circos package [69].

### 4.6. Nucleotide Substitution Rate Estimation

Phylosuite v1.2.2 [40] was used to locate and extract shared mitochondrial genes of the species. We used MAFFT v7.4 [70] and PhyloSuite v1.2.2 [67] to align and concatenate the nucleotide sequences. Pairwise nucleotide substitution rates, including nonsynonymous (dN) and synonymous (dS) substitution rates, as well as the dN/dS ratio, were calculated using the yn00 module in PAML v4.9 [72]. Visualization of the pairwise dN/dS values was achieved through box plots generated with the *ggplot2* package in R v4.2 [71].

## 5. Conclusions

In this study, the mitogenomes of *O. digyna* and *O. sinensis* were sequenced, assembled, annotated, and compared. Both *O. digyna* and *O. sinensis* exhibited notable structural polymorphism, characterized with linear and circular configurations. A long repeat sequence was identified and validated to mediate alternative conformations in the mitogenome of *O. sinensis*, enhancing our understanding of the structural diversity of mitogenome within Polygonaceae. Meanwhile, *O. digyna* and *O. sinensis* mitogenomes shared similar gene contents. The comparison of the mitochondrial and chloroplast genomes of *Oxyria* showed homologous regions and genes, suggesting potential gene transfer events between these organellar genomes. Phylogenetic analysis based on mitochondrial genes from eight selected Polygonaceae taxa suggests that the dataset can serve as a useful resource for phylogenetic studies. However, broader sampling may be required for more comprehensive conclusions. The analysis of mitochondrial gene composition across eight Polygonaceae species showed a mix of conservation and variability among PCGs, rRNA, and tRNA. This study provides a potential genomic background for better understanding and utilizing medicinal herbs.

## Figures and Tables

**Table 1:** Gene composition of the *O. digyna* and *O. sinensis* mitogenomes.

	Group of Genes	*O. digyna* Mitogenome		*O. sinensis* Mitogenome	
		Chromosome 1	Chromosome 2	Chromosome 1	Chromosome 2
Core genes	Complex I (NADH dehydrogenase)	*nad*1, *nad*2, *nad*3, *nad*4 *, *nad*5, *nad*4L, *nad*6, *nad*7 *, *nad*9		*nad*1, *nad*2, *nad*3, *nad*4 *, *nad*4L, *nad*5, *nad*6, *nad*7 *, *nad*9	
	Cytochrome c biogenesis	*cob*		*cob*	
	Complex IV (cytochrome c oxidase)	*cox*1, *cox*2	*cox*3	*cox*1, *cox*2 (2), *cox*3	
	ATP synthase	*atp*4, *atp*6, *atp*9	*atp*1, *atp*8	*atp*1, *atp*4, *atp*6, *atp*8,	*atp*9
	Maturases	*mat*R		*mat*R (2)	
	Cytochrome c biogenesis	*ccm*B, *ccm*C, *ccm*FC *, *ccm*FN		*ccm*B, ccmC, *ccm*FC *, *ccm*FN	
	Transport membrane protein	*mtt*B		*mtt*B	
Variable genes		*rpl*5, *rpl*16		*rpl*5, *rpl*16	
	Ribosomal protein	*rps*1, *rps*3 *, *rps*4, *rps*7, *rps*12, *rps*13 *rps*14		*rps*1, *rps*3 *, *rps*4, *rps*7, *rps*12, *rps*13, *rps*14	
	Complex II (succinate dehydrogenase)		*sdh*4	*sdh*4	
rRNA genes	Ribosomal RNAs	*rrn*5, *rrn*18	*rrn*26	*rrn*18, *rrn*26, *rrn*5	
tRNA	Transfer RNAs	*trn*C-GCA, *trn*E-CUC, *trn*E-UUC, *trn*F-GAA, *trn*G-GCC, *trn*H-GUG, *trn*K-UUU, *trn*M-CAU (5), *trn*N-GUU, *trn*P-UGG, *trn*Q-UUG, *trn*S-GCU, *trn*S-GGA (2), *trn*Y-GUA	*trn*A-UGC, *trn*D-GUC, *trn*H-GUG, *trn*L-UAA, *trn*P-UGG, *trn*S-GGA, *trn*W-CCA	*trn*A-UGC, *trn*C-GCA, *trn*D-GUC, *trn*E-CUC, *trn*E-UUC, *trn*F-GAA, *trn*G-GCC, *trn*H-GUG, *trn*K-UUU, *trn*L-UAA, *trn*M-CAU (5), *trn*N-GUU, *trn*P-UGG, *trn*Q-UUG, *trn*S-GCU, *trn*S-GGA (2), *trn*V-GAC, *trn*W-CCA	*trn*E-UUC (2), *trn*L-CAA, *trn*M-CAU, *trn*S-GAA

* Labeled intron containing genes, and bracketed numbers represent copy number of each gene.

## Data Availability

The chloroplast genome accession numbers for *O. digyna* and *O. sinensis* in GenBank are PP151304 and PP151305, respectively. The mitogenome accession numbers of *O. digyna* are PQ301199 and PQ301200. The mitogenome accession numbers of *O. sinensis* are PQ320132 and PQ320133. The sequencing reads used for mitogenomes assembly in this study have been released on the NCBI with the accession numbers PRJNA1157214 (BioProject), SAMN43508666 and SAMN43508667 (BioSample), and SRR30599217 and SRR30599218 (SRA). The sample was deposited in the Wuhan Botanical Garden with the accession numbers zhsl-2023 and sl-2023.

## Supplementary Materials

The following supporting information can be downloaded at https://www.mdpi.com/article/10.3390/ijms252211930/s1.
